# Short-term lifestyle education on obesity reduction in adolescents

**DOI:** 10.3389/fmed.2024.1308190

**Published:** 2024-03-25

**Authors:** Feng Ning, Xiaohui Sun, Bing Ge, Shunping Li, Binghui Hou, Yumei Wang, Dong Zhang

**Affiliations:** ^1^Department of Community Health, Qingdao Centers for Disease Control and Prevention, Qingdao, China; ^2^Department of Chronic Disease Prevention, Qingdao Centers for Disease Control and Prevention, Qingdao, China; ^3^Department of Neurology, The Affiliated Hospital of Qingdao University, Qingdao, China; ^4^Department of Disinfection Supply, The Qingdao 6th People’s Hospital, Qingdao, China; ^5^Department of Chronic Disease Prevention, Huangdao Centers for Disease Control and Prevention, Qingdao, China

**Keywords:** health education, community intervention, obesity, adolescent, prevalence

## Abstract

**Backgrounds:**

Obesity is increasing in adolescents in China. However, the awareness of obesity and prevention on related risk factors were not well known. We aim to assess the effectiveness of short-term health education intervention on obesity in Chinese adolescents.

**Methods:**

In this study, 42 primary and secondary schools from Qingdao were randomly divided into the education and control groups. A total of 11,739 adolescents was included in the current study. The logistic regression was employed to assess odds ratio (OR) of education intervention on overweight and obesity prevalence adjusting for covariates.

**Results:**

The baseline prevalence of overweight and obesity was significantly higher in urban than in rural areas and in boys than in girls. After 1 year lifestyle intervention, the proportion of students with awareness of obesity was higher, meanwhile age-adjusted mean values of weight, body mass index, duration of watching TV and doing homework were lower in education group than control group. The corresponding figures were 43.6 [95% CI (confidence intervals); 43.3–43.9] kg versus 44.3 (95% CI; 44.0–44.6) kg, 18.6 (95% CI; 18.5–18.7) kg/m^2^ versus 18.9 (95% CI; 18.8–19.1) kg/m^2^, 1.3 (95% CI; 1.2–1.3) hours/d versus 1.4 (95% CI; 1.3–1.4) hours/d, and 1.5 (95% CI; 1.4–1.5) hours/d versus 1.8 (95% CI, 1.7–1.8) hours/d. The multivariable adjusted OR for combined prevalence of overweight and obesity was 0.85 (95% CI, 0.76–0.96) in education group as compared with control group.

**Conclusion:**

Short-term health education intervention results in significantly higher reductions in obesity parameters and improvement in awareness in Chinese adolescents.

## Introduction

Emerging evidence has indicated the increasing prevalence of obesity in children and adolescents worldwide (1). With rapid urbanization and the introduction of western lifestyle, the prevalence of overweight and obesity in Chinese boys significantly increased from 1.57% and 0.35% in 1985 to 10.40% and 6.82% in 2005, respectively. The corresponding rates in girls increased from 1.74% and 0.31% in 1985 to 6.42% and 3.96% in 2005 (2). Meanwhile, the overall prevalence of type 2 diabetes among adolescents was 0.48 per 1,000 in Shanghai in 2005 and 2.80 per 1,000 in Tianjin in 2010, respectively (3, 4). Similarly, the overall prevalence in American adolescents increased from 0.34 per 1,000 in 2001 to 0.46 per 1,000 in 2009 (5). Data from the China Health and Nutrition Survey indicated that the standardized prevalence of hypertension of children and adolescent aged 9 to 17 years increased significantly from 8.08% in 2011 to 11.46% in 2015. Longitudinal studies have suggested that the increased prevalence of type 2 diabetes among the youth is attributed to the increase in obesity in pediatric populations (6, 7).Overall, overweight and obesity was widely demonstrated as the independent risk factor of adolescents’ type 2 diabetes and hypertension. Obesity or overweight in adolescents without sustainable and effective intervention, will dramatically increase risk for academic and cognitive achievement in adolescence and project to metabolic disorders and cardiovascular diseases mortality in later life. Therefore, obesity management at early stage is beneficial and economic approach tailored for adolescents development.

Both genetic modifications and potential environmental risk factors contributed to development of obesity. Currently, it is unclear which modifiable health behaviors are contributing to the vast increase in child and adolescent obesity. The emerging evidence on obesity pandemic is significantly associated with decline in physical activity, lower socioeconomic status and academic pressure in adolescent, but the conclusion is controversial. Data from the National Health and Nutrition Examination Survey indicated that only white adolescents reported an increasing prevalence of sports participation, slight declines in sports participation were observed in black or African American, Hispanic/Latino, and other adolescents. The Hong Kong adolescents study also mentioned the small declines in mean 9-min run/walk and sit-ups performance from 1998 to 2015, are suggestive of corresponding declines in cardiorespiratory fitness. The China Health and Nutrition Survey based on 2011 and 2015, showed that the prevalence of hypertension grew continuously in both genders, but the pace of change for boys was more rapid than that for girls. A meta-analysis demonstrated that the lower socioeconomic status and poorer mental health was decreased the adherence to obesity management among adolescents, with the pooled odds ratio of 1.34 [95%CI (confidence intervals); 1.19–1.52] and 1.12 (95% CI; 1.08–1.17), respectively. Health dietary patterns and calorie balance was widely recommended as weight management and cardiovascular disease prevention. Adherence to dietary guidelines seems to be a protective factor for obesity in adults, but this relationship is not confirmed in adolescents.

Several systematic reviews have shown that school-based prevention of obesity is feasible and effective, but the conclusions were not consistent. Most of the interventions were applied for a school-based approach, and all interventions emphasized the importance of having a healthy diet, active lifestyle and normal body weight, however, the parental involvement and social environment was not fully evaluated. The previous study has been unconvincing evidence on school-based programs to reduce any measure of overweight and obesity and awareness of obesity prevention and health lifestyle in adolescent. The effectiveness of school-based nutrition interventions may also change by socioeconomic status, culture and different conditions of adolescent’s growth and development. The purpose of the Qingdao Adolescence Diabetes Education Program was to evaluate the effectiveness of 1 year intervention combined with school-based and family involvement education on the risk of overweight and obesity among adolescents in China.

## Method

### Study population

Qingdao Adolescence Diabetes Education Program involves the implementation of a school-based survey for obesity risk intervention. A stratified, random cluster sampling method was used to recruit a representative sample of adolescents aged 10 to 18 years. Two urban (Shinan and Shibei districts) and two rural (Huangdao and Jiaonan cities) areas were randomly selected from the 12 districts of Qingdao. All enrolled schools were randomly allocated to either the education or control group. A total of 14,000 participants were randomly selected from 21 primary and 21 secondary schools. Health education and promotion were completed in the education schools, whereas no such intervention was conducted in the control counterparts. Students were eligible for participation if they did not have diabetes or any serious physical condition that would preclude regular participation in physical education. The adolescents and guardians refused to enter the cohort or without informed consent was excluded in the current study. A total of 12,638 individuals participated in the follow-up examination after 1 year. We control the sample replacement rate of current study at less than 5% and with a participate rate of 90.3%. Participants with missing variables such as height or weight measurement or other information at the end of follow up were excluded from the current analysis. Finally, a total of 11,739 individuals with available information were included in the current study. Written consent was obtained from adolescents and their guardians before the surveys. The study was approved by the Ethics Committee of Qingdao Centers for Disease Control and Prevention.

### Intervention

School physicians and nurses delivered health promotion and education to students and guardians through pamphlets and lectures in the education group. The educational intervention consisted of three components: (a) Classroom curriculum, (b) School environment support and (c) Parental involvement. As for classroom education, school physicians delivered physical education and healthy diet education to students, for instance, the knowledge of obesity, diabetes and healthy dietary of 6 grams salt intake daily. The school environmental activities included the healthy education curriculum three times per week, calorie intake recommended by the guideline in student canteens, slogan posted on the corridor and playground, and physical activity three more time than usual curriculum referred by the Education Administration. The healthy education included food labels reading behavior and nutritional guideline, regular sleep, psychological course, risk and prevention informations on obesity and chronic diseases. The physical activity included football, volleyball, basketball, table-tennis, running and so on. Meanwhile, we posted or updated periodically the healthy lifestyle slogans in the blackboards or playground in the intervention school. The series of health information was also transferred to their parents with health promotion named as “small hand connect to big hand.” The health coordinator encouraged guardians and adolescents to purchase healthy snacks and food both on the way to and from school. The education schools reduced the amount of unhealthy foods in student canteens, and they discouraged parents from buying sugar-added and high-caloric foods and encouraged them to replace these foods with high-fiber foods for their children during summer and winter breaks. Along with booklets, some salt spoons and oil scaled tools were delivered to family kitchens to limit the salt and oil consumption. The above campaign was launched to increase awareness of the recommended food intake, pay more attention to the labeling on processed foods, and take action to reduce sodium, sugar and oil intake in their diets.

Students were also suggested to meet the recommended daily guideline of 60 min of moderate to vigorous physical activity and the screen viewing time guidelines of 2 h or less daily, while students in the control schools followed their usual health practice and dietary pattern.

### Anthropometric measurements and questionnaires

Participants were interviewed by trained school physicians or nurses. Anthropometric measurements were obtained for body weight and height. Height and weight were measured with participants wearing light clothes but no shoes. Body mass index (BMI) was defined as body weight divided by the square of height (kg/m^2^). Overweight and obesity were stratified by age and sex according to the Study Group of Chinese Adolescent Obesity (8). Individuals with BMI between the 85th and 95th percentile for age and sex are considered overweight, and those with BMI at or above the 95th percentile are considered obese. The duration of watching TV, physical activity, and doing homework after the completion of intervention was also recorded.

The risk factors and protective factors for obesity were evaluated after 1 year follow-up. The studied risk factors were age, obesity, excess hamburger consumption, cola drinking, and family history of diabetes or hypertension. The protective factors for diabetes and obesity were the frequency of physical activity, housework, cereal intake, and vegetable consumption. A family history of diabetes was defined as having at least one parent or sibling with diabetes. The knowledge of clinical symptoms and treatments for diabetes was collected from adolescents. After 1 year follow-up, standard questionnaires related to risk factors and protective factors for obesity and diabetes were administered to individuals in the education and control schools.

### Statistical analyses

The means of obesity indicators and other continuous variables were calculated using the general linear model with adjustment for age at baseline. Analysis of variance was used to evaluate differences in categorical variables between the two groups. The Chi-square test was used to investigate the difference in proportions between the education and control groups. Multivariable adjusted logistic regression was employed to assess the relationship between the risk factors and obesity. The odds ratio (OR) and 95%CI for obesity were estimated to identify potential risk factors and protective factors. The potential covariates included age, gender, region, BMI, duration of watching TV, duration of doing homework, knowledge of obesity reduction. All statistical analyses were performed using SPSS, Version 21.0 (SPSS Inc., Chicago, IL, United States). A two-tailed *p* value of less than 0.05 was considered statistically significant.

## Result

A total of 11,739 adolescents were included in the current analysis. The prevalence of overweight and obesity was significantly higher in urban areas than in rural areas and in boys than in girls (12.8% vs. 6.9%, 14.1% vs. 7.8%, 11.0% vs. 6.0%, and 11.3% vs. 7.8%, respectively; *p* < 0.001 for all comparisons). However, the combined prevalence of overweight and obesity was not different between the education and control groups (18.4% vs. 17.7%; *p* = 0.267). The age-adjusted mean values of weight, BMI, duration of watching TV, and duration of doing homework were significantly lower in the education group than in the control group (*p* < 0.001 for all comparisons). The corresponding means and confidence intervals were 43.6 (95% CI; 43.3–43.9) kg versus 44.3 (95% CI; 44.0–44.6) kg, 18.6 (95% CI; 18.5–18.7) kg/m^2^ versus 18.9 (95% CI; 18.8–19.1) kg/m^2^, 1.3 (95% CI; 1.2–1.3) hours/d versus 1.4 (95% CI; 1.3–1.4) hours/d, and 1.5 (95% CI; 1.4–1.5) hours/d versus 1.8 (95% CI; 1.7–1.8) hours/d, respectively. Similar trends were also observed both in boys and girls. The duration of physical activity occurring outside was not significantly different between the two groups (Table 1).

**Table 1:** Characteristics of study population after 1 year follow-up.

	Education schools	Control schools	*p* values
No.	6,518	5,221	
Age (years)	12.1 (95%CI; 12.0–12.1)	12.6 (95%CI;12.5–12.6)	0.000
Gender, % (*n*)			
Male	50.1 (3267)	49.7 (2593)	
Female	49.9 (3251)	50.3 (2628)	0.622
Weight (kg)	43.6 (95%CI; 43.31–43.86)	44.2 (95%CI; 43.88–44.49)	0.005
BMI (kg/m^2^)	18.5 (95%CI; 18.42–18.65)	18.9 (95%CI; 18.75–19.00)	0.000
Watching TV (hours/d)	1.14 (95%CI; 1.10–1.15)	1.30 (95%CI;1.26–1.31)	0.000
Homework (hours/d)	1.43 (95%CI;1.40–1.45)	1.70 (95%CI;1.67–1.73)	0.000
Physical activity (hours/d)	1.86 (95%CI; 1.84–1.89)	1.85 (95%CI; 1.82–1.89)	0.172
Obesity status,% (*n*)			
Normal	81.3 (5297)	81.3 (4243)	
Overweight	9.2 (597)	8.4 (436)	
Obesity	9.6 (624)	10.4 (542)	0.132
Do you have knowledge of diabetes? % (*n*)			
Yes	94.9 (6185)	82.4 (4300)	
No	3.4 (223)	9.7 (506)	
I have no idea	1.7 (110)	7.9 (415)	0.000
Do you know family history of diabetes will increase risk for diabetes? % (*n*)			
Yes	71.4 (4655)	32.6 (1701)	
No	6.9 (452)	9.3 (483)	
I have no idea	21.6 (1411)	58.2 (3037)	0.000
Do you know the relationship between hypertension, cardiac diseases and diabetes? % (*n*)			
Yes	62.9 (4099)	30.6 (1597)	
No	18.4 (1201)	17.6 (917)	
I have no idea	18.7 (1218)	51.8 (2707)	0.000
Do you know obesity increase risk of diabetes? % (*n*)			
Yes	91.5 (5966)	67.8 (3539)	
No	1.7 (108)	2.5 (130)	
I have no idea	6.8 (444)	29.7 (1552)	0.000
Do you know relationship between physical activity and risk of diabetes? % (*n*)			
Increase	0.8 (53)	1.0 (50)	
Decrease	93.9 (6122)	74.5 (3891)	
I have no idea	5.3 (343)	24.5 (1280)	0.000
Do you know relationship between doing housework and risk of diabetes? % (*n*)			
Increase	2.6 (172)	4.4 (231)	
Decrease	85.8 (5593)	51.8 (2703)	
I have no idea	11.6 (753)	43.8 (2287)	0.000
Do you know vegetable intake reduce risk of diabetes? % (*n*)			
Yes	94.8 (6179)	88.7 (4631)	
No	5.2 (339)	11.3 (590)	0.000
Do you know cereal intake reduce risk of diabetes? % (*n*)			
Yes	85.6 (5577)	66.5 (3474)	
No	14.4 (941)	33.5 (1747)	0.000
Do you know eating hamburger increase risk of diabetes? % (*n*)			
Yes	98.9 (6447)	96.9 (5059)	
No	1.1 (71)	3.1 (162)	0.000
Do you know drinking cola increase risk of diabetes? % (*n*)			
Yes	99.4 (6478)	97.9 (5111)	
No	0.6 (40)	2.1 (110)	0.000

Data are expressed as age-adjusted mean (95%confidence intervals) and percentage (number). BMI, body mass index.

The proportion of students with awareness of obesity, diabetes, and their risk factors was significantly higher in the education group than in the control group after 1 year follow-up (*p* < 0.001 for all comparisons). For example, awareness of vegetable and fruit consumption, cereal intake, and the duration of physical activity and doing housework were associated with the reduction in the risks of obesity and type 2 diabetes, and increase in age, hamburger consumption, and a family history of diabetes were associated with increased risks (*p* < 0.001 for all comparisons).

As listed in Table 2, ORs for the combined prevalence of overweight and obesity significantly decreased with age and in rural residents, girls, and high education level in model 1. The corresponding ORs remained stable after further adjustment in model 3.The multivariable adjusted OR for combined prevalence of overweight and obesity was 0.85 (95% CI; 0.76–0.96) in education group as compared with control group. In addition, the duration of watching TV and hamburger consumption were significantly associated with the increased prevalence of overweight and obesity. However, the duration of physical activity and vegetable and fruit consumption did not reduce the risk of overweight and obesity in a multivariable model.

**Table 2:** Odds ratios (95% confidence intervals) for prevalence of overweight and obesity in multivariable adjusted models.

	Model1	Model 2	Model 3	Change in -2Log Likelihood(df = 1)	Significance of the Change
Age(years)	0.71 (95%CI; 0.69–0.73)	0.71 (95%CI; 0.69–0.73)	0.72(95%CI; 0.70–0.74)	559.879	0.000
Residential area (rural vs. urban)	0.67 (95%CI; 0.60–0.74)	0.67 (95%CI; 0.60–0.75)	0.63(95%CI; 0.56–0.71)	59.422	0.000
Gender (female vs. male)	0.54 (95%CI; 0.49–0.60)	0.55 (95%CI; 0.50–0.61)	0.55(95%CI; 0.50–0.61)	139.279	0.000
Education	0.75 (95%CI; 0.68–0.84)	0.78 (95%CI; 0.70–0.87)	0.85(95%CI; 0.76–0.96)	7.218	0.007
Watching TV(hours/d)	–	1.21 (95%CI; 1.15–1.27)	1.19(95%CI; 1.13–1.24)	44.427	0.000
Homework (hours/d)	–	1.00 (95%CI; 0.95–1.06)	0.99(95%CI; 0.94–1.05)	0.050	0.824
Physical activity (hours/d)	–	0.96 (95%CI; 0.92–1.00)	0.97(95%CI; 0.92–1.01)	2.642	0.104
Do you know physical activity reduce risk of diabetes?	–	–			
Yes vs. No	–	–	0.65 (95%CI; 0.40–1.05)		
I do not know vs. No	–	–	0.85 (95%CI; 0.51–1.39)	11.154 (2)	0.004
Do you know doing housework reduce risk of diabetes?	–	–			
Yes vs. No	–	–	1.27 (95%CI; 0.95–1.68)		
I do not know vs. No	–	–	1.28 (95%CI; 0.95–1.72)	2.958 (2)	0.228
Do you know vegetable intake reduce risk of diabetes?	–	–	0.93 (95%CI; 0.77–1.13)	0.503	0.478
Do you know cereal intake reduce risk of diabetes?	–	–	0.88 (95%CI; 0.77–1.00)	3.971	0.046
Do you know eating hamburger increase risk of diabetes?	–	–	1.65 (95%CI; 1.15–2.36)	7.237	0.007
Do you know drinking cola increase risk of diabetes?	–	–	0.97 (95%CI; 0.62–1.53)	0.016	0.900

Data are expressed as odds ratios (95% confidence intervals) or other representations in the table. DF, degree of freedom; NA, not available.

In a sensitivity analysis, the conclusions were consistent in the two age group of 10–13 and 14–18 years. For instance, children living in rural areas, and girl was associated with reduced risk for overweight and obesity in both age groups. Compared with the control group, the education group significantly reduced risk for overweight and obesity in older group, moderate in younger group, the corresponding OR and 95%CIs were 0.75 (95% CI, 0.58–0.97) and 0.89 (95% CI, 0.78–1.01), respectively. The increased frequency of physical activity, vegetable consumption and age were associated with obesity reduction in older group, whereas the duration of watching TV and hamburger consumption increased risk for overweight and obesity in younger group.

## Discussion

We found that short-term school-based education intervention was associated with a 15% decrease risk for prevalence of overweight and obesity. The average weight and BMI were significantly lower in the education group than in the control group, whereas education intervention moderately affect the combined prevalence in adolescent aged 10 to 13. Compared with the control schools, individuals in the education schools had more knowledge and awareness of overweight and obesity risks and their protective factors and more participation in physical activity after short-term intervention.

Several studies have reported the impact of school based education interventions on students’ obesity and diabetes problems, however, failed to reach to consistent conclusions. Recently research revealed that school-based education interventions were ineffective on reducing the BMI increment in primary school students (9), while some others held the opposite conclusion (10, 11). Some potential study characteristics, such as study population, age, study design, intervention duration, strategies, should consider to explain the inconsistency of the conclusions (12). With the introduction of western lifestyles and rapid urbanization, the combined prevalence of overweight and obesity has increased dramatically in Chinese adolescents during the last three decades (13). Overweight or obesity is associated with high blood pressure, insulin resistance, and high C-reactive protein and lipid levels in Chinese adolescents, independent of risk factors for type 2 diabetes, metabolic syndrome, and other complications in later life (14). Without effective lifestyle intervention and a health education strategy, the rapid urbanization of China is expected to sustain the trends.

The obesity reduction and awareness improvement was notable in girls than in boys. As for the gender differences on overweight and obesity in the current study, was consistent with national level studies in China. Generally, the prevalence of overweight and obesity is higher in boys than in girls. It confirmed these trends in different time periods, with great disparities between boys and girls, irrespective of age, location, and economic status. Considering the traditional attitudes on feeding boys and girls differently, Chinese parents distribute more high-calorie food to their boys. Meawhile, girls in adolescence are more discipline and easier to follow the intervention advice, and as well as to keep slim and beautiful to control the energy intake. Therefore, the short-term education and awareness of obesity reduction is more remarkable in girls than in boys. Based on national surveys in China, the prevalence of overweight and obesity among young individuals is higher in urban than in rural areas. In particular, the average annual increase in overweight and obesity among rural children exceeded that among their urban peers. Remarkably, the increase trend for obesity in rural areas was more faster than in urban counterparts both in the current adolescent cohort and adult cohort in Qingdao. This increase in urbanization and decline in physical activity, may be the main factor of the gradually decreasing differences between urban and rural areas. Across three decades, school-aged children and adolescents in China have increased in BMI, with less disparity between urban and rural locations. The family history of obesity or diabetes of parents in rural areas may positive deliver intervention messages to their child, because of chronic disease burden and intervention benefit. The hypothesis should be tested in the further analysis.

A sedentary and unhealthy lifestyle is significantly associated with the risks of obesity and other metabolic disorders. Our study supported that the duration of watching TV was significantly associated with the prevalence of overweight and obesity. Similarly, a meta-analysis including 232 studies of 983,840 participants indicated that watching TV for more than 2 h per day was associated with unfavorable body composition and low fitness levels (15). In particular, the duration of watching TV is associated with reduced physical and psychosocial health; thus, lowering sedentary time leads to a reduction in BMI. In addition, sedentary behavior is significantly associated with school achievement and mental health indicators (16–19).

A family clustering and twins study showed that environmental factors play a significant role in the development of obesity and diabetes. A previous study indicated that a high proportion of parents and grandparents underestimate their child’s weight status, particularly those in three-generation families. These misconceptions and the unhealthy diet may partially contribute to the overweight or obesity epidemic among Chinese adolescents (20, 21). However, information on the guardians’ attitude and socioeconomic status was not available to assess the association with metabolic disorders. Generally, among adolescents, the intake of most of their total calories occurs outside school, and food from home and roadside snacks are major contributors. Studies have demonstrated that a community-based environmental change in childhood obesity intervention influenced parents, resulting in reduced parental BMI. In addition, reading the nutrition labels was associated with the reduction in overall energy and fat intake in adolescents (22, 23). Further research should examine the effects of different types of interventions on parental and community-setting health behaviors and health outcomes.

Inconsistent results have been obtained for the effectiveness of short-term lifestyle intervention on overweight or obesity and weight change in adolescents (15, 24–32). In a meta-analysis including several randomized clinical trials, compared with the control arm, the ORs for overweight and obesity in the school-based intervention programs indicated that the intervention was significantly protective [0.74 (95%CI; 0.60–0.92) kg], without an effective decrease in BMI [−0.62 (95%CI; −1.39–0.14) kg/m^2^] (27). Another study including 9,750 adolescents from six Chinese urban cities demonstrated that the school-based integrated intervention was cost effective for improving the BMI of school children but had resulted in borderline significant reduction in overweight and obesity prevalence between the combined physical and dietary intervention and control groups (30). In our study, OR for the combined prevalence of overweight and obesity was 0.85 (95% CI; 0.76–0.96) kg for the education group as compared with the control group. Our study demonstrated that school-based education reduces the risk of obesity and diabetes. However, the reasonability and stability of early intervention in school settings require further investigation.

The effectiveness of lifestyle intervention should be investigated throughout real-life settings in the future. Another Chinese study including 2,425 children showed that the prevalence of overweight and obesity was significantly lower in the intervention schools than in the control schools after 3-year intervention (26.3 and 32.5%, respectively). The mean BMI was significantly lower in the intervention schools than in the control schools (28). Our study indicated that age-adjusted means of weight and BMI were significantly lower in the education schools than in the control schools [43.6 (95%CI; 43.3–43.9) kg vs. 44.3 (95% CI; 44.0–44.6) kg and 18.6 (95% CI; 18.5–18.7) kg/m^2^ vs. 18.9 (95% CI; 18.8–19.1) kg/m^2^, respectively]. These observations highlight that early intervention in overweight and obese adolescents may reduce the development of these metabolic risk factors. However, the long-term effects and cost effectiveness of lifestyle intervention on obesity in adolescents must be evaluated in additional studies. Future research should translate this positive information into clinical practice guidelines and disseminate this information to healthcare providers and the general public.

With the implementation of the “Healthy China 2030 Plan” and the enhancement of family health awareness, the National Health Committee launched the Obesity Prevention and Control Demonstration Action among primary and secondary school students in Qingdao and other cities in China. The school nutrition promotion and action findings were also discussed and distributed in international forum and local school-based nutrition policy. Recently, the Qingdao local government also distribute food subsidies as well as nutritional programs including school feeding to meet the criteria of energy intake. With the help of health and educational authorities, we establish cooperation between health and educational sections to integrate intervention into schools’ regular academic programs. Thus, the annual health and nutritional status of students monitored the results of nutritional advice and obesity prevention, and modify the conventional policy accordingly.

The current study has some strengths. The study design was prospective with cluster random sample selection. The definitions of overweight and obesity for Chinese adolescents were based on international standards. However, this study also has some limitations. Some students did not participate in the field survey and fill the questionnaires. We controlled the sample replacement rate of current study at less than 5% and participate rate in a reasonable level. Moreover, the baseline information of the missing participants was not significantly different from counterparts included in the current study. We obtained information on baseline weight and height from school physicians, and the mean obesity indicators were not significantly different between the education and control schools at baseline. The effect of education on the change in weight could not be evaluated at the end of this study. Moreover, the effect of family’s cooking habit, dietary patterns, and socioeconomic status was not addressed in the current study. The Food Frequency Questionnaire and total energy intake was not available and cannot adjust in the current data analysis. Moreover, the amount reduction on unhealthy foods in student canteens or outside school retail stores was also not collected. We will carry out the Food Frequency Questionnaire in the further investigation. The efficacy study should be cautious to generalize the feasibility, effectiveness, or sustainability of an intervention program outside a study setting. The preliminary outcomes require further investigation.

## Conclusion

In conclusion, the combined prevalence of overweight and obesity was not significantly reduced in the education schools after short-term intervention. However, the intervention resulted in significant reductions in various indicators of adiposity and increased awareness of obesity. Short-term school-based lifestyle education may reduce the risk of obesity in children and adolescents. The effectiveness of school-based education on obesity intervention requires further investigation.

## Data availability statement

The original contributions presented in the study are included in the article/supplementary material, further inquiries can be directed to the corresponding author.

## Ethics statement

The studies involving humans were approved by Ethics Committee of Qingdao Centers for Disease Control and Prevention. The studies were conducted in accordance with the local legislation and institutional requirements. Written informed consent for participation in this study was provided by the participants’ legal guardians/next of kin.

## Author contributions

FN: Formal analysis, Funding acquisition, Writing – original draft. XS: Investigation, Writing – review & editing. BG: Data curation, Investigation, Writing – original draft. SL: Investigation, Methodology, Writing – original draft. BH: Formal analysis, Methodology, Writing – review & editing. YW: Conceptualization, Supervision, Writing – review & editing. DZ: Conceptualization, Project administration, Supervision, Writing – review & editing.
